# *Treponema pallidum* Induces the Secretion of HDVSMC Inflammatory Cytokines to Promote the Migration and Adhesion of THP-1 Cells

**DOI:** 10.3389/fcimb.2019.00220

**Published:** 2019-06-21

**Authors:** Zheng-Xiang Gao, Li-Li Liu, Li-Rong Lin, Man-Li Tong, Fan Liu, Tian-Ci Yang

**Affiliations:** ^1^Center of Clinical Laboratory, Zhongshan Hospital, School of Medicine, Xiamen University, Xiamen, China; ^2^Institute of Infectious Disease, School of Medicine, Xiamen University, Xiamen, China

**Keywords:** *Treponema pallidum*, human dermal vascular smooth muscle cells, adherence, migration, cytokine

## Abstract

The pathological features of syphilis, a disease caused by *Treponema pallidum* (*T. pallidum*), are characterized by vascular involvement with endarteritis and periarteritis. Little is known about the interactions of infiltrating immunocytes with human dermal vascular smooth muscle cells (HDVSMCs) in arterioles during the immunopathogenesis of syphilis. In the present study, we demonstrated that stimulation of HDVSMCs with *T. pallidum* resulted in the upregulated gene transcription and protein expression of interleukin (IL)-6, monocyte chemoattractant protein-1 (MCP-1), and intercellular adhesion molecule-1 (ICAM-1) in a dose- and time-dependent manner. Moreover, the migration and adhesion of THP-1 cells to HDVSMCs were significantly suppressed by anti-MCP-1 and anti-ICAM-1 neutralizing antibodies, respectively. Further studies revealed that *T. pallidum* activated the NF-κB signaling pathway in HDVSMCs. Inhibition of NF-κB suppressed *T. pallidum*-induced IL-6, MCP-1, and ICAM-1 expression. In addition, the migration and adhesion of THP-1 cells to *T. pallidum*-treated HDVSMCs were significantly decreased by pretreatment with an NF-κB inhibitor. These findings demonstrate that *T. pallidum* induces the production of IL-6, MCP-1, and ICAM-1 in HDVSMCs and promotes the adherence and migration of THP-1 cells to HDVSMCs through the NF-κB signaling pathway, which may provide new insight into the pathogenesis of *T. pallidum* infection.

## Introduction

Syphilis is a severe sexually transmitted disease caused by *Treponema pallidum* subsp. pallidum (*T. pallidum*), a gram-negative bacterium from the spirochete family with a circular DNA genome lacking metabolic and viral genes. Although drug treatment is inexpensive and effective, syphilis remains a worldwide public health problem as a chronic infectious disease (LaFond and Lukehart, 2006; Bellhouse et al., 2018). The pathological features of syphilis are characterized by vascular involvement with endarteritis and periarteritis (Singh and Romanowski, 1999). In primary syphilis, the inflammatory infiltrate is predominantly composed of lymphocytes and plasma cells, although macrophages are also observed in lesions, and a single chancre forms at the site of inoculation. In secondary syphilis, a wide variety of histological changes occur, and *T. pallidum* infection leads to variable systemic symptoms characterized by vascular inflammation and increased angiogenesis. The typical symptom of syphilis is a generalized skin rash (Baughn and Musher, 2005; LaFond and Lukehart, 2006). In these two stages, *T. pallidum* has been observed by electron microscopy to be in direct contact with smooth muscle cells of dermal arterioles (Martin-Ezquerra et al., 2009).

Vascular inflammation is a complex and multifactorial pathophysiological process that plays a crucial role in the development and progression of disease (Clarke et al., 2010; Iwata et al., 2010; Yang et al., 2018; Zeng et al., 2018). Vascular smooth muscle cells (VSMCs) are the main cellular component of the middle vascular layer, which is not a passive bystander during vascular inflammation. For example, in addition to leukocytes, VSMCs could be another crucial source of inflammatory cytokines in the vessel wall (Chen et al., 1998; Kranzhofer et al., 1999). Studies have demonstrated that various pathogenic factors induce the expression of inflammatory cytokines in VSMCs; identified factors include vascular cell adhesion molecule-1 (VCAM-1), chemokines such as monocyte chemotactic protein-1 (MCP-1) and interleukin (IL)-8, and inflammatory markers such as IL-1β, IL-6, and tumor necrosis factor-α (TNF-α) (Takaguri et al., 2011; Wakabayashi and Takeda, 2013; Fan et al., 2016; Zeng et al., 2018). Thus, VSMCs play a key role in vascular inflammation related to functional and organic disorders (Tohru et al., 2003; Yang et al., 2018).

Previous studies have shown that *T. pallidum* and its membrane lipoprotein can induce the expression of adhesion factors in human vascular endothelial cells and promote the adhesion of inflammatory cells to human vascular endothelial cells (Riley et al., 1992, 1994; Zhang et al., 2014, 2015). No evidence, however, has been provided about the role of VSMCs in vascular inflammation in syphilis. Therefore, we postulate that *T. pallidum* can induce the expression of inflammatory cytokines and promote the adherence of immunocytes to VSMCs in human dermal arterioles, which may be important for the immunopathogenesis of syphilis. Here, we aimed to demonstrate whether *T. pallidum* is capable of inducing the production of inflammatory cytokines and the activation of relevant signaling pathways in human dermal VSMCs (HDVSMCs) and to evaluate the influence of *T. pallidum* on the migration and adherence of human monocytic cells (THP-1) to HDVSMCs.

## Materials and Methods

### Cell Culture

HDVSMCs (purchased from CH Scientific, Inc., Boston, MA, USA) were incubated in DMEM/F-12 medium supplemented with 20% (v/v) fetal bovine serum (Biological Industries Ltd., Kibbutz Beit HaEmek, Israel). After the HDVSMCs grew to confluence, quiescence was induced by incubation in serum-free DMEM/F-12 for 24 h, and the quiescent cells were used in the following experiments. The cytotoxicity assay was performed using a lactate dehydroge- nase (LDH) kit according to the manufacturer's instructions (NEOBIOSCIENCE Biotechnology Co., Ltd. Beijing, China).

### Expression of Inflammatory Cytokines in HDVSMCs Stimulated by *T. pallidum*

The *T. pallidum* Nichols strain was kindly provided by Lorenzo Giacani, PhD (University of Washington, Seattle, WA, USA) and propagated in rabbits as previously described (Tong et al., 2017). HDVSMCs were incubated with *T. pallidum* at different multiplicities of infection (MOIs, *T. pallidum:* cell ratios of 1:1, 10:1, 100:1, and 200:1) for 24 h. Following treatment, the mRNA levels of IL-1β, IL-6, MCP-1, intercellular adhesion molecule-1 (ICAM-1) and TNF-α were detected by quantitative real-time PCR (qRT-PCR) as described previously (Su et al., 2018; Jiang et al., 2019). Briefly, total RNA was isolated from the cells using an RNA Extraction Kit (TIANGEN Biotechnology Beijing Co., Ltd.) and then reverse transcribed with a high-capacity cDNA reverse transcription kit (Takara, Shiga, Japan). qRT-PCR was carried out using QuantiFast SYBR one-step RT-PCR (Qiagen, Shanghai, China) and the LightCycler®96 instrument (Roche Diagnostics, Roche Instrument Center AG, Rotkreuz, Switzerland). The results were calculated based on fold differences relative to the level of glyceraldehyde 3-phosphate dehydrogenase (GAPDH) by the 2^−ΔΔCT^ method. Then, HDVSMCs were incubated with *T. pallidum* at an MOI of 100:1 for different durations (0, 3, 6, 12, 24, 48, and 72 h). The mRNA levels of IL-1β, IL-6, MCP-1, ICAM-1, and TNF-α were measured by qRT-PCR. The primers used in the qRT-PCR analyses are listed in Table 1.

**Table 1:** Primers used for qRT-PCR analysis in this study.

**Gene**	**Oligonucleotide primer sequences (5**^****′****^**-3**^****′****^**)**	
IL-1β	Forward	TGGCAATGAGGATGACTTGT
	Reverse	TGGTGGTCGGAGATTCGTA
IL-6	Forward	TACATCCTCGACGGCATCTC
	Reverse	TTTCAGCCATCTTTGGAAGG
MCP-1	Forward	CATTGTGGCCAAGGAGATCTG
	Reverse	CTTCGGAGTTTGGGTTTGCTT
ICAM-1	Forward	CTCCAATGTGCCAGGCTTG
	Reverse	CAGTGGGAAAGTGCCATCCT
TNF-α	Forward	CTTCTCGAACCCCGAGTGAC
	Reverse	ATGAGGTACAGGCCCTCTGA
GADPH	Forward	GAAGGTGAAGGTCGGAGTC
	Reverse	GAAGATGGTGATGGGATTTC

Cell culture supernatants were used to assess IL-6 and MCP-1 concentrations with commercial ELISA kits (eBioscience, San Diego, USA) according to the manufacturer's protocols. The ICAM-1 protein levels in the HDVSMCs were assessed by western blot as described previously (Yang et al., 2018). In addition, to determine the percentage of ICAM-positive cells, the HDVSMCs were incubated with a PE Mouse Anti-Human ICAM-1 antibody (BD Biosciences, San Diego, USA) according to the manufacturer's protocols, and the fresh HDVSMC suspension was immediately analyzed by fluorescence-activated cell sorting (FACS). The mean fluorescence intensity (MFI) of ICAM-1 was detected using a FACSCanto II flow cytometer (BD Biosciences, San Diego, USA). The results were analyzed using FlowJo 7.6.4 software (Tree Star, Ashland, OR, USA).

### Analysis of NF-κB Activation in *T. pallidum*-Treated HDVSMCs

HDVSMCs were incubated with *T. pallidum* at an MOI of 100:1 for various durations. Cell lysates were collected, and the levels of phosphorylated and total IκBα protein (Cell Signaling Technology, Danvers, MA, USA) were assessed by western blot. To further confirm that *T. pallidum* induced the activation of the NF-κB signaling pathway, we pre-incubated HDVSMCs with a 2 μmol/L concentration of the NF-κB inhibitor BAY11-7082 (Sigma-Aldrich, St. Louis, MO, USA) for 1 h and then incubated the cells with *T. pallidum* at an MOI of 100:1 for 30 min. Subsequently, we assessed the levels of phosphorylated and total IκBα protein. In addition, cells were stimulated as described above for 24 h to determine the expression levels of IL-6, MCP-1, and ICAM-1.

### Analysis of NF-κB p65 Subunit Translocation Into the Nucleus

HDVSMCs were cultivated in a Millicell EZ Slide 4-well glass slide box (Millipore, Burlington, MA, USA) and stimulated with *T. pallidum* at an MOI of 100:1 for 30 min. Then, the translocation of the NF-κB p65 subunit into the nucleus was analyzed as described previously (Park et al., 2015). The MFI of the NF-κB p65 subunit in the nucleus was detected by ImageJ. For the inhibition assay, HDVSMCs were pretreated with 2 μmol/L BAY11-7082 for 1 h and then incubated with *T. pallidum* for 30 min. NF-κB p65 subunit translocation into the nucleus was analyzed as described above.

### Cell Migration Assays

Cell migration assays were performed using 24-well Transwell plates (Corning Costar, Pittston, PA). First, HDVSMCs were pretreated with *T. pallidum* at different MOIs (*T. pallidum*: cell ratios of 1:1, 10:1, 100:1 and 200:1) at 37°C in an atmosphere containing 5% CO_2_ for 24 h in 24-well culture plates. Then, 600 μL of cell culture supernatant was collected and added to the lower chambers of Transwell plates. Approximately 2.5 ×10^5^ THP-1 cells in a volume of 200 μL were seeded in the upper chambers of the Transwell plates. The Transwell plates were then incubated at 37°C in an atmosphere containing 5% CO_2_ for 2 h. THP-1 cells that migrated to the lower chamber were counted under a microscope, and the migration rate of THP-1 cells was calculated as follows: migration rate of THP-1 cells (%) = (number of THP-1 cells in the lower chamber/number of THP-1 cells added to the upper chamber) ×100. For the inhibition assays, HDVSMCs were pre-incubated with an anti-MCP-1 neutralizing antibody (30 μg/mL) (R&D Systems, Inc., Minneapolis, MN, USA) or BAY11-7082 (2 μmol/L) for 1 h. Subsequently, HDVSMCs were incubated with *T. pallidum* at an MOI of 100:1 for 24 h. HDVSMCs treated with phosphate-buffered saline (PBS) were used as the control cells. The migration assay was performed, and the migration of THP-1 cells was calculated as described above.

### Adherence Assay

HDVSMCs were treated with *T. pallidum* at different MOIs (*T. pallidum*: cell ratios of 1:1, 10:1, 100:1, and 200:1) at 37°C in an atmosphere containing 5% CO_2_ for 24 h. THP-1 cells were stained with 10 μM calcein AM at 37°C for 30 min, added to 1 ×10^5^ THP-1 cells and incubated at 37°C for 1 h. After the cells were washed with RPMI-1640 medium three times, adhered THP-1 cells were observed by fluorescence microscopy and counted by ImageJ software. For the experiments involving pharmacological inhibitors, HDVSMCs were pre-incubated with an anti-ICAM-1 neutralizing antibody (10 μg/mL) (R&D Systems, Inc., Minneapolis, MN, USA) or BAY11-7082 (2 μmol/L) for 1 h and then incubated with *T. pallidum* at an MOI of 100:1 for 24 h. HDVSMCs treated with PBS were used as the control cells, and the adherence assay was performed as described above.

### Statistical Analysis

All data in the present study are expressed as the means ± SDs. To compare values among multiple groups, one-way ANOVA was applied. A 2-sided *P* < 0.05 was considered statistically significant. All statistical analyses were performed using SPSS 22.0 for Windows (SPSS Inc., Chicago, IL, USA).

## Results

### *T. pallidum* Induced the mRNA Expression of IL-6, MCP-1, and ICAM-1 in HDVSMCs

**Figure 1:**
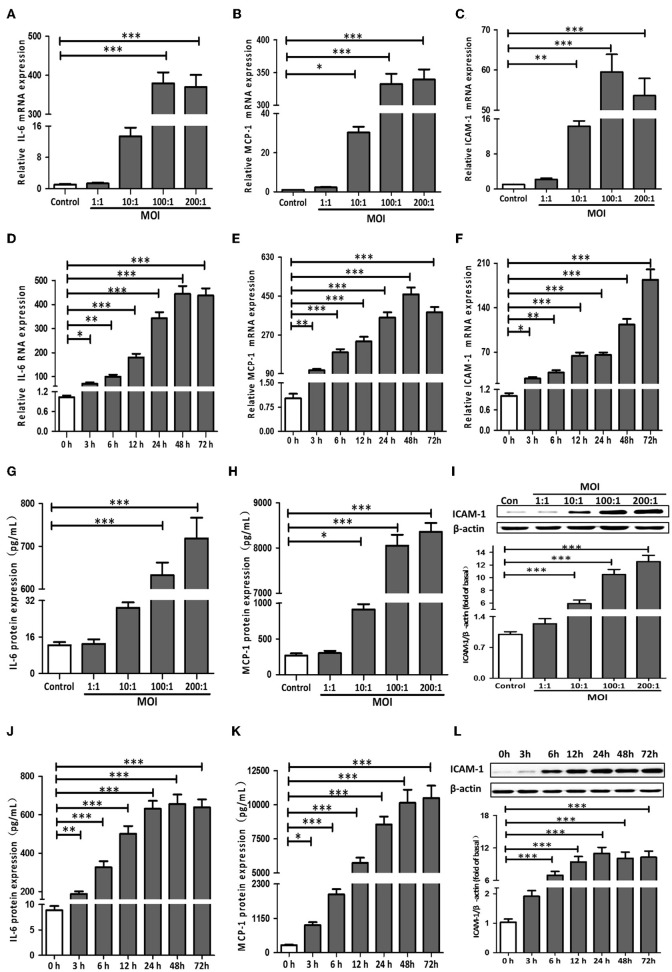
*T. pallidum* induced the expression of IL-6, MCP-1, and ICAM-1 in HDVSMCs. The HDVSMCs were incubated with *T. pallidum* at different MOIs for 24 h or at an MOI of 100:1 for different amounts of time. The mRNA expression was evaluated by qRT-PCR. The levels of soluble IL-6 and MCP-1 were evaluated by ELISA. The protein expression of ICAM-1 was detected by western blot. **(A,D)** The mRNA expression of IL-6. **(B,E)** The mRNA expression of MCP-1. **(C,F)** The mRNA expression of ICAM-1. **(G,J)** The protein expression of soluble IL-6. **(H,K)** The protein expression of soluble MCP-1. **(I,L)** The protein expression of ICAM-1. The values are the means ± SDs of experimental triplicates and are representative of the results of three independent experiments. IL-6, interleukin-6; ICAM-1, intercellular cell adhesion molecule*-*1; MCP-1, monocyte chemotactic protein-1; MOI, multiplicity of infection (^*^P < 0.05, ^**^P < 0.01, ^***^P < 0.001).

First, we detected the mRNA expression levels of IL-1β, TNF-α, IL-6, MCP-1, and ICAM-1 in HDVSMCs incubated with *T. pallidum* at different MOIs. As shown in Figures 1A–C, *T. pallidum* markedly increased the mRNA expression of IL-6, MCP-1 and ICAM-1 in a dose-dependent manner. The mRNA expression of IL-6 was significantly increased at an MOI of 100:1 (*P* < 0.001) and peaked at an MOI of 200:1 (*P* < 0.001) (Figure 1A). The mRNA expression of MCP-1 was significantly increased at an MOI of 10:1 (*P* < 0.05) and peaked at an MOI of 200:1 (*P* < 0.001) (Figure 1B). The mRNA expression of ICAM-1 was significantly increased at an MOI of 10:1 (*P* < 0.01), peaked at an MOI of 100:1 (*P* < 0.001), and subsequently decreased at an MOI of 200:1 (Figure 1C). The mRNA expression of IL-1β and TNF-α was not significantly changed at different MOIs (Figures S1A,B).

Meanwhile, HDVSMCs were incubated with *T. pallidum* at an MOI of 100:1 for 0, 3, 6, 12, 24, 48, and 72 h. *T. pallidum* markedly increased the mRNA expression of IL-6, MCP-1 and ICAM-1 in a time-dependent manner (Figures 1D–F). The mRNA expression level of IL-6 was significantly increased at 3 h (*P* < 0.05), and the maximal level was attained within 48 h (*P* < 0.001) (Figure 1D). The MCP-1 mRNA expression level was significantly increased at 3 h (*P* < 0.01), peaked within 48 h (*P* < 0.001), and decreased at 72 h (Figure 1E). The ICAM-1 mRNA expression level was significantly increased at 3 h (*P* < 0.05) and peaked by 72 h (*P* < 0.001) (Figure 1F). The mRNA expression of IL-1β and TNF-α was not significantly changed at the different time points (Figures S1C,D).

### *T. pallidum* Induced the Protein Expression of IL-6, MCP-1, and ICAM-1 in HDVSMCs

To investigate the effects of *T. pallidum* on the protein expression of IL-6, MCP-1, and ICAM-1 in HDVSMCs, we incubated HDVSMCs with *T. pallidum* at MOIs of 1:1, 10:1, 100:1, and 200:1 for 24 h. *T. pallidum* significantly promoted the protein expression of IL-6, MCP-1, and ICAM-1 in a dose-dependent manner. The IL-6 secretion was significantly increased at an MOI of 100:1 (*P* < 0.001) and peaked at an MOI of 200:1 (*P* < 0.001) (Figure 1G). The level of soluble MCP-1 was significantly increased at an MOI of 10:1 (*P* < 0.05) and peaked at an MOI of 200:1 (*P* < 0.001), indicating a concentration-dependent response (Figure 1H). ICAM-1 protein expression was detected by western blot and flow cytometry. The protein expression of ICAM-1 and the MFI of ICAM-1 was significantly increased at an MOI of 10:1 and peaked at an MOI of 200:1, indicating a dose-dependent response (Figure 1I and Figure S2A). IL-1β and TNF-α secretion were not significantly changed at the different MOIs (Figures S1E,F).

In addition, HDVSMCs were incubated with *T. pallidum* at an MOI of 100:1 for 0, 3, 6, 12, 24, 48, and 72 h. *T. pallidum* markedly increased the protein expression of IL-6, MCP-1, and ICAM-1 in a time-dependent manner. The level of soluble IL-6 was significantly increased at 3 h (*P* < 0.01) and peaked at 48 h (*P* < 0.001) (Figure 1J). The level of soluble MCP-1 was significantly increased at 3 h (*P* < 0.05) and peaked at 48 h (*P* < 0.001) (Figure 1K). The protein expression of ICAM-1 was significantly increased at 6 h and peaked at 24 h (*P* < 0.001) (Figure 1L). The MFI of ICAM-1 was significantly increased at 6 h (*P* < 0.05) and peaked at 48 h (*P* < 0.001) (Figure S2B). The IL-1β and TNF-α secretion were not significantly changed at the different time points (Figures S1G,H).

### NF-κB Signaling Pathway Components Were Essential for the Induction of IL-6, MCP-1, and ICAM-1 Expression by *T. pallidum*

**Figure 2:**
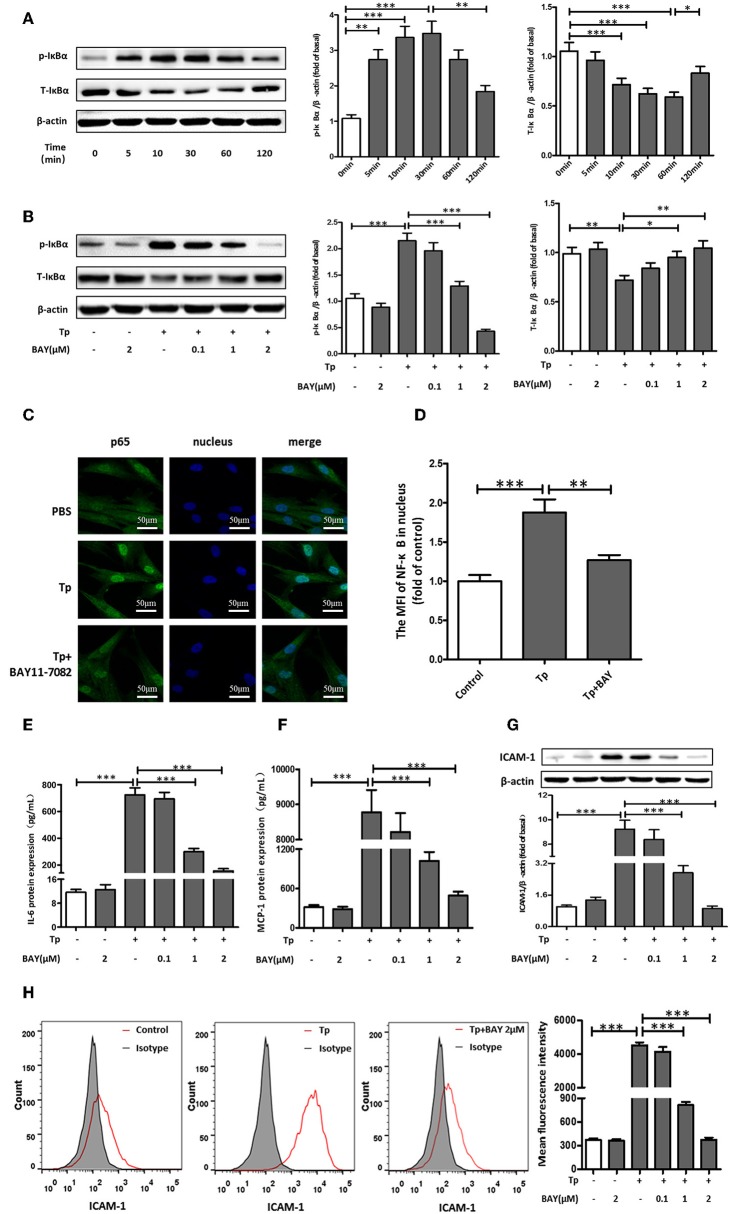
NF-κB signaling pathway components were essential for the induction of IL-6, MCP-1, and ICAM-1 expression by *T. pallidum*. **(A)** The HDVSMCs were incubated with *T. pallidum* at an MOI of 100:1 for different amounts of time, the levels of phosphorylated and total IκBα protein were detected by western blot. **(B)** The HDVSMCs were pretreated with BAY11-7082 for 1 h and then incubated with *T. pallidum* at an MOI of 100:1 for 30 min, the levels of phosphorylated and total IκBα protein were detected by western blot. **(C)** The translocation of the NF-κB p65 subunit into the nucleus (600×). **(D)** The MFI of the NF-κB p65 subunit in the nucleus was detected by ImageJ. **(E–H)** HDVSMCs were pretreated with BAY11-7082 (2 μmol/L) for 1 h and then incubated with *T. pallidum* at an MOI of 100:1 for 24 h. The levels of soluble IL-6 and MCP-1 were evaluated by ELISA. The protein expression of ICAM-1 was detected by western blot. The MFI of ICAM was detected by flow cytometry. **(E)** The protein expression of soluble IL-6. **(F)** The protein expression of soluble MCP-1. **(G)** The protein expression of ICAM-1. **(H)** The MFI of ICAM-1. The values are the means ± SDs of experimental triplicates and are representative of the results of three independent experiments. Tp, *T. pallidum*; IL-6, interleukin-6; ICAM-1, intercellular cell adhesion molecule*-*1; MCP-1, monocyte chemotactic protein-1; BAY, BAY11-7082; MFI, mean fluorescence intensity; μM, μmol/L (^*^P < 0.05, ^**^P < 0.01, ^***^P < 0.001).

To determine whether NF-κB was involved in *T. pallidum*-induced IL-6, MCP-1, and ICAM-1 expression in HDVSMCs, we examined the phosphorylation of IκBα, a well-defined indicator of NF-κB activation. IκBα is a regulatory protein that inhibits NF-kB activity, and its phosphorylation and subsequent degradation leads to NF-kB activation/nuclear translocation. The IκBα phosphorylation stimulated by *T. pallidum* in a time-dependent manner exhibited a significant increase at 5 min (*P* < 0.01), peaked at 30 min (*P* < 0.001), and declined at 120 min (*P* < 0.01) (Figure 2A). In addition, the total IκBα level decreased starting at 10 min but recovered at 120 min. Moreover, the phosphorylation of IκBα was inhibited by BAY11-7082 (NF-κB inhibitor) in a concentration-dependent manner at 30 min (Figure 2B).

To further verify that NF-κB was involved in the induction of IL-6, MCP-1, and ICAM-1 expression by *T. pallidum*, we used an immunofluorescence-based approach. Treatment with *T. pallidum* induced a significant increase in the NF-κB immunofluorescence signal in the nucleus, indicating the apparent translocation of NF-κB from the cytoplasm to nuclear areas of *T. pallidum*-treated HDVSMCs. This phenomenon was inhibited by pretreatment with BAY11-7082 (Figures 2C,D). Similarly, pretreatment with BAY11-7082 attenuated *T. pallidum*-induced IL-6, MCP-1 and ICAM-1 mRNA expression (Figures S3A–C) and protein expression (Figures 2E–H and Figure S3D) in a concentration-dependent manner at 24 h.

### *T. pallidum* Promoted the Adherence of THP-1 Cells to HDVSMCs by Modulating MCP-1 and ICAM-1 Expression

**Figure 3:**
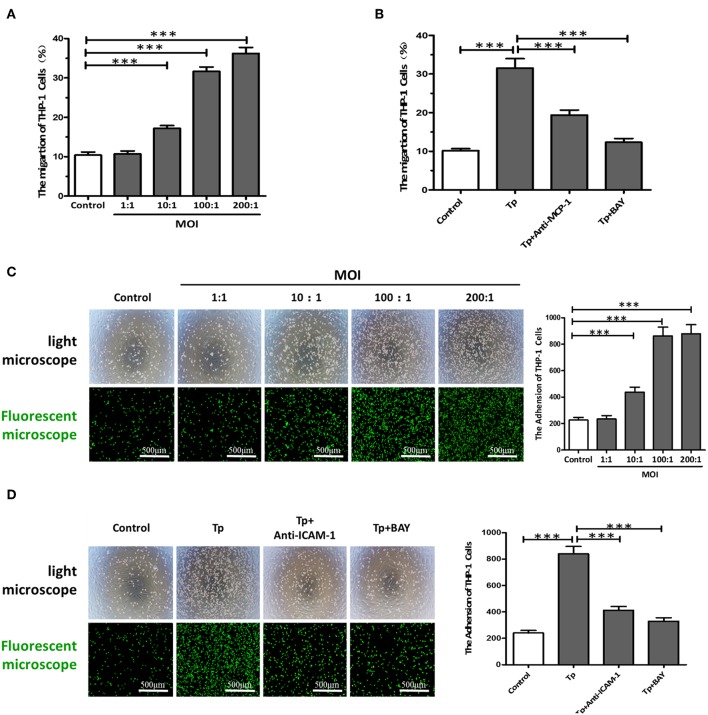
*T. pallidum* promotes the adherence of THP-1 cells to HDVSMCs by modulating MCP-1 and ICAM-1 expression. **(A)** The migration of THP-1 cells to HDVSMCs was induced by *T. pallidum*. **(B)** Both an anti-MCP-1 neutralizing antibody (30 μg/mL) and BAY11-7082 (2 μmol/L) prevented *T. pallidum*-induced THP-1 cell migration. The data shown are the percentages of total cells. **(C)** The adhesion of THP-1 cells to HDVSMCs (100×); the upper panels were observed by light microscopy, and the lower panels were observed by fluorescence microscopy. **(D)** Both an anti-ICAM-1 neutralizing antibody (10 μg/mL) and BAY11-7082 (2 μmol/L) prevented *T. pallidum*-induced HDVSMC adhesion (100×). The values are the means ± SDs of experimental triplicates and are representative of the results of three independent experiments. Tp, *T. pallidum*; BAY, BAY11-7082; MOI, multiplicity of infection (^***^P < 0.001).

The migration of immune cells to the site of inflammation is an early event in vascular inflammation. To examine the chemotaxis of monocytes toward HDVSMCs, we added THP-1 cells to the inserts of a Transwell system containing cell culture medium harvested from cultures of HDVSMCs pretreated with *T. pallidum*. As shown in Figure 3A, *T. pallidum* stimulated an increase in the migration of THP-1 cells to HDVSMCs. The migration rate was significantly increased at an MOI of 10:1 (*P* < 0.001) and peaked at an MOI of 200:1 (*P* < 0.001). To determine whether MCP-1 plays an important role in the chemotactic activity of THP-1 cells, we used an anti-MCP-1 neutralizing antibody to block MCP-1. The THP-1 cell migration rate significantly decreased under these conditions (*P* < 0.001). Furthermore, pretreatment of HDVSMCs with BAY11-7082 attenuated the *T. pallidum*-induced migration of THP-1 cells at 2 h (Figure 3B).

To examine the effects of *T. pallidum* on the adhesion of THP-1 cells to HDVSMCs, we pretreated HDVSMCs with *T. pallidum* at different MOIs (*T. pallidum*: cell ratios of 1:1, 10:1, 100:1, and 200:1) for 24 h, followed by incubation with THP-1 cells for 1 h at 37°C. *T. pallidum* noticeably increased the adhesion of THP-1 cells to HDVSMCs in a dose-dependent manner (Figure 3C). The number of adhered cells was significantly increased at an MOI of 10:1 (*P* < 0.001) and peaked at an MOI of 100:1 (*P* < 0.001). To identify the cell surface molecules that mediate the adhesion of THP-1 cells to HDVSMCs, we used an anti-ICAM-1 neutralizing antibody to block ICAM-1. The adherence of THP-1 cells to HDVSMCs was significantly inhibited (*P* < 0.001). Furthermore, the adhesion of THP-1 cells was attenuated by pretreatment of the HDVSMCs with BAY11-07082 before incubation with *T. pallidum* (*P* < 0.001) (Figure 3D).

## Discussion

*T. pallidum* secretes no exotoxins, and the lipopolysaccharide on its outer membrane does not have endotoxin activity. Therefore, the tissue damage and primary clinical symptoms caused by syphilis arise from the host inflammatory response. In addition, compared to the pathogenic mechanisms of other bacterial pathogens, the pathogenic mechanisms of *T. pallidum* are not well-understood (Ho and Lukehart, 2011). Vascular inflammation is mainly mediated by a variety of inflammatory molecules, many of which are secreted by vascular endothelial cells and smooth muscle cells (Minamino et al., 2003; Satoh et al., 2008). VSMCs have also been shown to express adhesion molecules (e.g., VCAM-1 and ICAM-1) (Shin et al., 2016; Huang et al., 2017), chemokines (e.g., MCP-1) and cytokines (e.g., IL-6 and IL-8) (Wakabayashi and Takeda, 2013). Numerous *T. pallidum* bacteria are reportedly arranged predominately in a perivascular pattern, which is defined as a “vasculotropic pattern” (Martin-Ezquerra et al., 2009). A previous study demonstrated that *T. pallidum* at MOIs from 100:1 to 1000:1 could activate human vascular endothelial cells and promote the expression of ICAM-1 *in vitro* (Riley et al., 1992). In the present study, incubating HDVSMCs with *T. pallidum* at MOIs from 10:1 to 200:1 led to the significant upregulation of IL-6, MCP-1 and ICAM-1 expression. In addition, *T. pallidum* promoted the migration and adherence of THP-1 cells to HDVSMCs, indicating that HDVSMCs could play an important role in *T. pallidum*-induced vascular inflammation.

IL-6 is a pleiotropic cytokine that plays a predominant role in various inflammatory diseases (Erta et al., 2012; Mihara et al., 2012). Cells known to express IL-6 include all stromal cells and cells of the immune system. In addition, IL-6 is considered to be a better predictor of disease activity than C-reactive protein (Vincenzo et al., 2004; Fraunberger et al., 2006). *T. pallidum* lipoproteins and flagellin have been reported to increase the expression of IL-6 in monocytes and may play an important role in the inflammatory process in syphilis (Liu et al., 2010; Xie et al., 2017). In this study, *T. pallidum* increased the expression of IL-6 in HDVSMCs in a concentration- and time-dependent manner. Moreover, IL-6 is important in the transition from acute inflammation to either acquired immunity or chronic inflammatory disease. IL-6 dysregulation contributes to chronic inflammation in certain conditions (Gabay, 2006; Hunter and Jones, 2015) Therefore, we hypothesize that overproduction of IL-6 plays a crucial role in the inflammatory responses caused by *T. pallidum*, thus contributing to the pathogenesis of syphilis.

Studies have demonstrated that the interaction of VSMCs with leukocyte cells through adhesion molecules and chemokines is a crucial event in vascular inflammatory reactions (Bishop-Bailey et al., 1998; Montecucco and Mach, 2009). Interactions between VSMCs and transmigrated leukocytes induce pro-inflammatory responses and vascular dysfunction (Zhu et al., 2000; Cai et al., 2004). MCP-1, which belongs to the CC chemokine family, was recognized as playing an important role in the migration and activation of monocytes (Deshmane et al., 2009). The recombinant *T. pallidum* proteins Tp0965 and Tp17 can activate vascular endothelial cells and increase MCP-1 secretion (Zhang et al., 2014, 2015). Here, we found that the MCP-1 gene transcription and level of MCP-1 in the culture supernatant were increased by *T. pallidum* stimulation in a time- and dose-dependent manner. In addition, the increased migration rate of THP-1 cells incubated with *T. pallidum* to HDVSMCs was significantly decreased by treatment with the anti-MCP-1 neutralizing antibody.

ICAM-1 is a member of the immunoglobulin superfamily (IGSF) of adhesion molecules that mediates the adhesion of monocytes to vascular cells. Increased expression of ICAM-1 on VSMCs can facilitate the accumulation of transmigrated leukocytes in the vascular walls of atherosclerotic lesions (Koo et al., 2015). Rhinovirus was shown to elevate the expression of ICAM-1 in human airway smooth muscle cells, which may play a role in virus-induced asthma exacerbations (Oliver et al., 2006). Another study found that ICAM-1 levels were elevated in the sera of *Chlamydia pneumoniae*-seropositive patients, which may underlie the mechanisms linking *C*. *pneumoniae* infection and atherosclerosis *in vivo* (Kohara et al., 2002). Here, we assessed the expression of ICAM-1 induced by *T. pallidum* in HDVSMCs and found that the expression of ICAM-1 was increased in a concentration- and time-dependent manner after incubation with *T. pallidum*. In addition, the adhesion of THP-1 cells to HDVSMCs, which was increased by *T. pallidum* stimulation, was inhibited by the anti-ICAM-1 neutralizing antibody. These results indicate that the increased expression of MCP-1 and ICAM-1 in HDVSMCs incubated with *T. pallidum* may play a crucial role in the *T. pallidum*-induced inflammatory response by recruiting immunocytes to sites of inflammation.

Inflammatory responses following exposure to a stimulator are highly dependent on the activation of the NF-κB transcription factor, which plays an important role in the immunoinflammatory response by modulating the complex network of effectors and cell signaling pathways (Hayden and Sankar, 2008). The main regulatory mechanism of this central transcription factor is via the phosphorylation of IκBα, which leads to the proteasome-mediated degradation of IκBα, ultimately resulting in the activation and nuclear translocation of NF-κB (Hayden and Sankar, 2008). Our study demonstrated that the increased p-IκBα levels and NF-κB translocation were inhibited by BAY11-7082. Furthermore, the *T. pallidum*-induced expression of IL-6, MCP-1 and ICAM-1 was significantly inhibited by BAY11-7082, suggesting that *T. pallidum*-stimulated IκBα phosphorylation and NF-κB translocation are essential for inflammatory factor upregulation and are involved in the migration and adhesion of THP-1 cells to HDVSMCs.

In this study, we investigated the role of *T. pallidum*-induced inflammatory factor secretion in HDVSMCs. Only certain inflammatory factors were analyzed in this study, and determining whether other inflammatory factors are secreted by HDVSMCs in *T. pallidum*-induced vascular inflammation will require further research. Furthermore, the NF-κB inhibitor BAY11-7082 used in our experiments might also affect other pathways, such as phosphatases, and we cannot completely exclude off-target effects. In addition, further *in vivo* studies are needed to confirm our *in vitro* findings.

In summary, our results support the hypothesis that *T. pallidum* plays a direct role in the induction of IL-6, MCP-1, and ICAM-1 production in HDVSMCs and promotes the adherence and migration of THP-1 cells to HDVSMCs. Moreover, this process is mediated by the NF-κB signaling pathway, which could contribute to the pathogenesis of *T. pallidum*.

## Data Availability

The raw data supporting the conclusions of this manuscript will be made available by the authors, without undue reservation, to any qualified researcher.

## Author Contributions

Z-XG and T-CY conceived and designed the experiments. Z-XG performed the experiments. L-RL, L-LL, and FL analyzed the data. Z-XG and M-LT contributed reagents, materials, and analysis tools. Z-XG and T-CY wrote the paper.

### Conflict of Interest Statement

The authors declare that the research was conducted in the absence of any commercial or financial relationships that could be construed as a potential conflict of interest.

## Supplementary Material

Figure S1 *T. pallidum* induced the protein expression of IL-1β and TNF-α in HDVSMCs at different MOIs or times. The HDVSMCs were incubated with *T. pallidum* at different MOIs for 24 h or at an MOI of 100:1 for different amounts of time. The mRNA expression was evaluated by qRT-PCR. The levels of soluble IL-1β and TNF-α were evaluated by ELISA. **(A,C)** The mRNA expression of IL-1β. **(B,D)** The mRNA expression of TNF-α. **(E,G)** The protein expression of soluble IL-1β. **(F,H)** The protein expression of soluble MCP-1. The values are the means ± SDs of experimental triplicates and are representative of the results of three independent experiments. IL-1β, interleukin-1β; TNF-α, tumor necrosis factor-α; MOI, multiplicity of infection; NS, no significance.

Figure S2 Flow cytometric analysis of how *T. pallidum* induced the expression of ICAM-1 in HDVSMCs at different MOIs or times. The MFI of ICAM was detected by flow cytometry. **(A)** HDVSMCs were incubated with *T. pallidum* at different MOIs for 24 h. **(B)** HDVSMCs were incubated with *T. pallidum* at an MOI of 100:1 for different amounts of time. The values are the means ± SDs of experimental triplicates and are representative of the results of three independent experiments. ICAM-1, intercellular cell adhesion molecule*-*1; MFI, mean fluorescence intensity (^*^*P* < 0.05, ^***^*P* < 0.001).

Figure S3 NF-κB signaling pathway components were essential for the induction of IL-6, MCP-1, and ICAM-1 expression by *T. pallidum*. HDVSMCs were pretreated with BAY11-7082 (2 μmol/L) for 1 h and then incubated with *T. pallidum* at an MOI of 100:1 for 24 h. The mRNA expression was evaluated by qRT-PCR. The MFI of ICAM was detected by flow cytometry. **(A)** The mRNA expression of IL-6. **(B)** The mRNA expression of MCP-1. **(C)** The mRNA expression of ICAM-1. **(D)** The MFI of ICAM-1. The values are the means ± SDs of experimental triplicates and are representative of the results of three independent experiments. Tp, *T. pallidum*; ICAM-1, intercellular cell adhesion molecule*-*1; BAY, BAY11-7082; μM, μmol/L; MFI, mean fluorescence intensity (^***^*P* < 0.001).
