# Supplementary figures and images for: Treponema pallidum Induces the Secretion of HDVSMC Inflammatory Cytokines to Promote the Migration and Adhesion of THP-1 Cells
Source: Front Cell Infect Microbiol. 2019 Jun 21;9:220. doi: 10.3389/fcimb.2019.00220 (PMC6598120; doi:10.3389/fcimb.2019.00220)

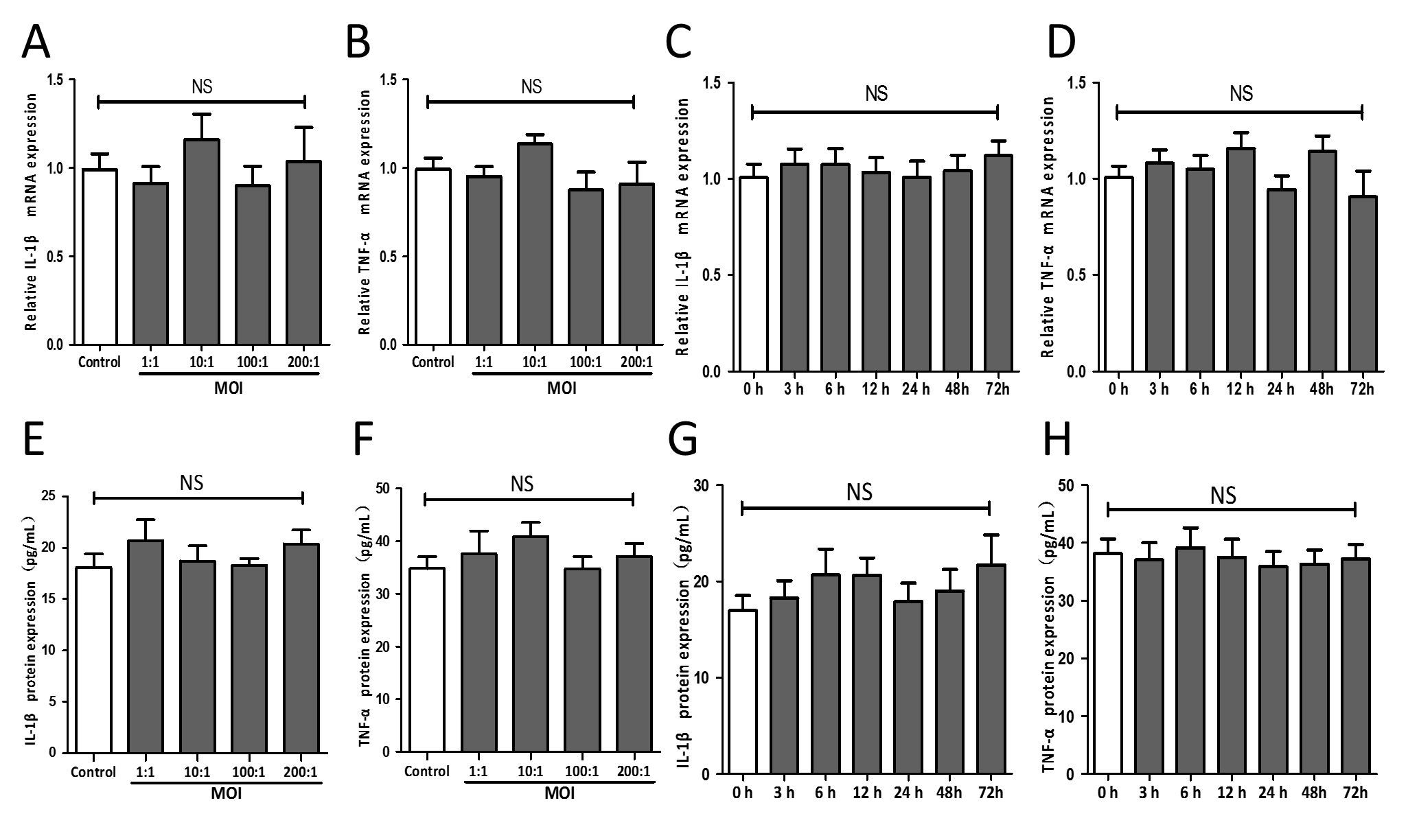

Supplement: Figure S1 — T. pallidum induced the protein expression of IL-1β and TNF-α in HDVSMCs at different MOIs or times. The HDVSMCs were incubated with T. pallidum at different MOIs for 24 h or at an MOI of 100:1 for different amounts of time. The mRNA expression was evaluated by qRT-PCR. The levels of soluble IL-1β and TNF-α were evaluated by ELISA. (A,C) The mRNA expression of IL-1β. (B,D) The mRNA expression of TNF-α. (E,G) The protein expression of soluble IL-1β. (F,H) The protein expression of soluble MCP-1. The values are the means ± SDs of experimental triplicates and are representative of the results of three independent experiments. IL-1β, interleukin-1β; TNF-α, tumor necrosis factor-α; MOI, multiplicity of infection; NS, no significance. [file Image_1.TIF]

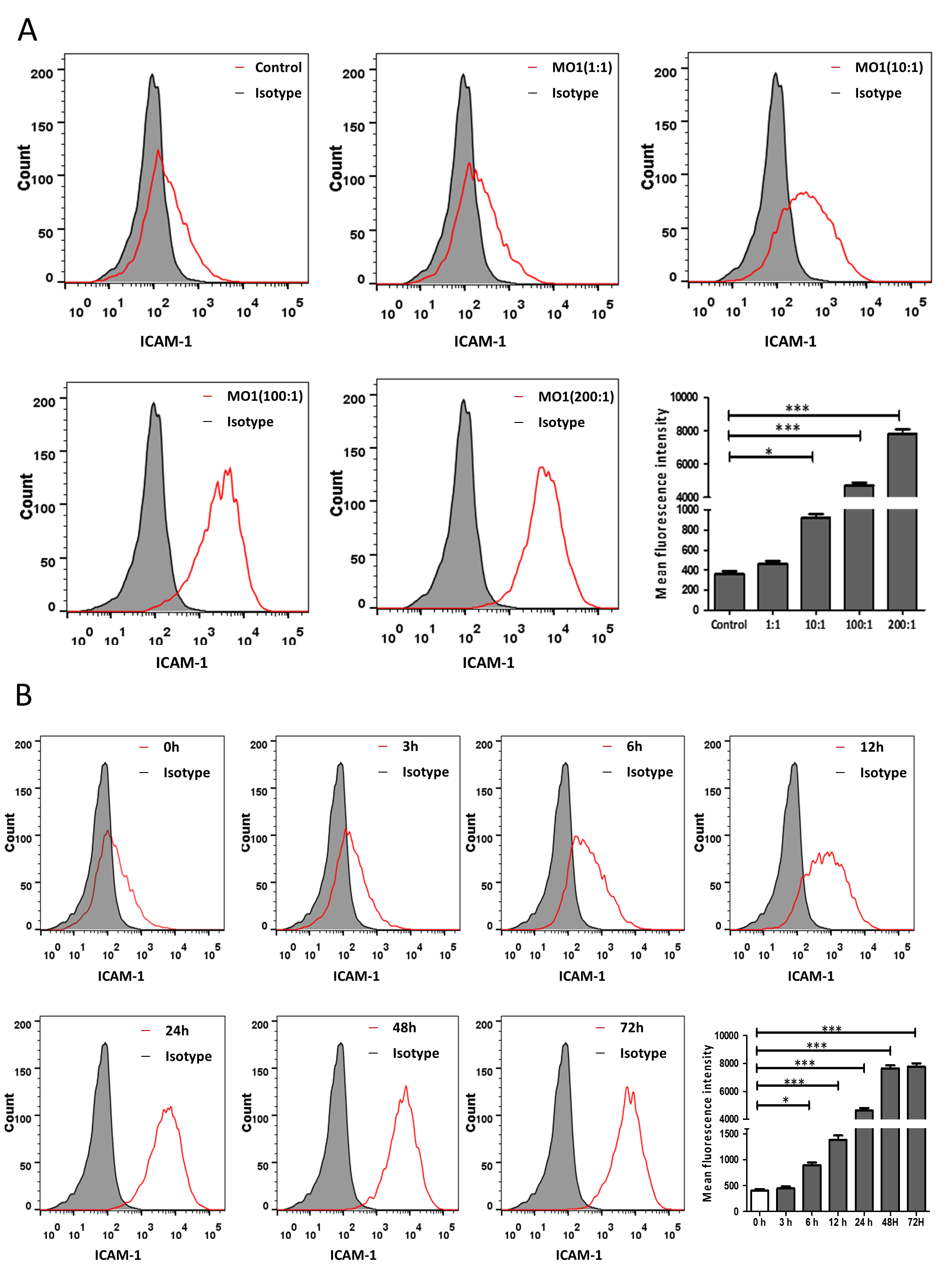

Supplement: Figure S2 — Flow cytometric analysis of how T. pallidum induced the expression of ICAM-1 in HDVSMCs at different MOIs or times. The MFI of ICAM was detected by flow cytometry. (A) HDVSMCs were incubated with T. pallidum at different MOIs for 24 h. (B) HDVSMCs were incubated with T. pallidum at an MOI of 100:1 for different amounts of time. The values are the means ± SDs of experimental triplicates and are representative of the results of three independent experiments. ICAM-1, intercellular cell adhesion molecule-1; MFI, mean fluorescence intensity (*P < 0.05, ***P < 0.001). [file Image_2.TIF]

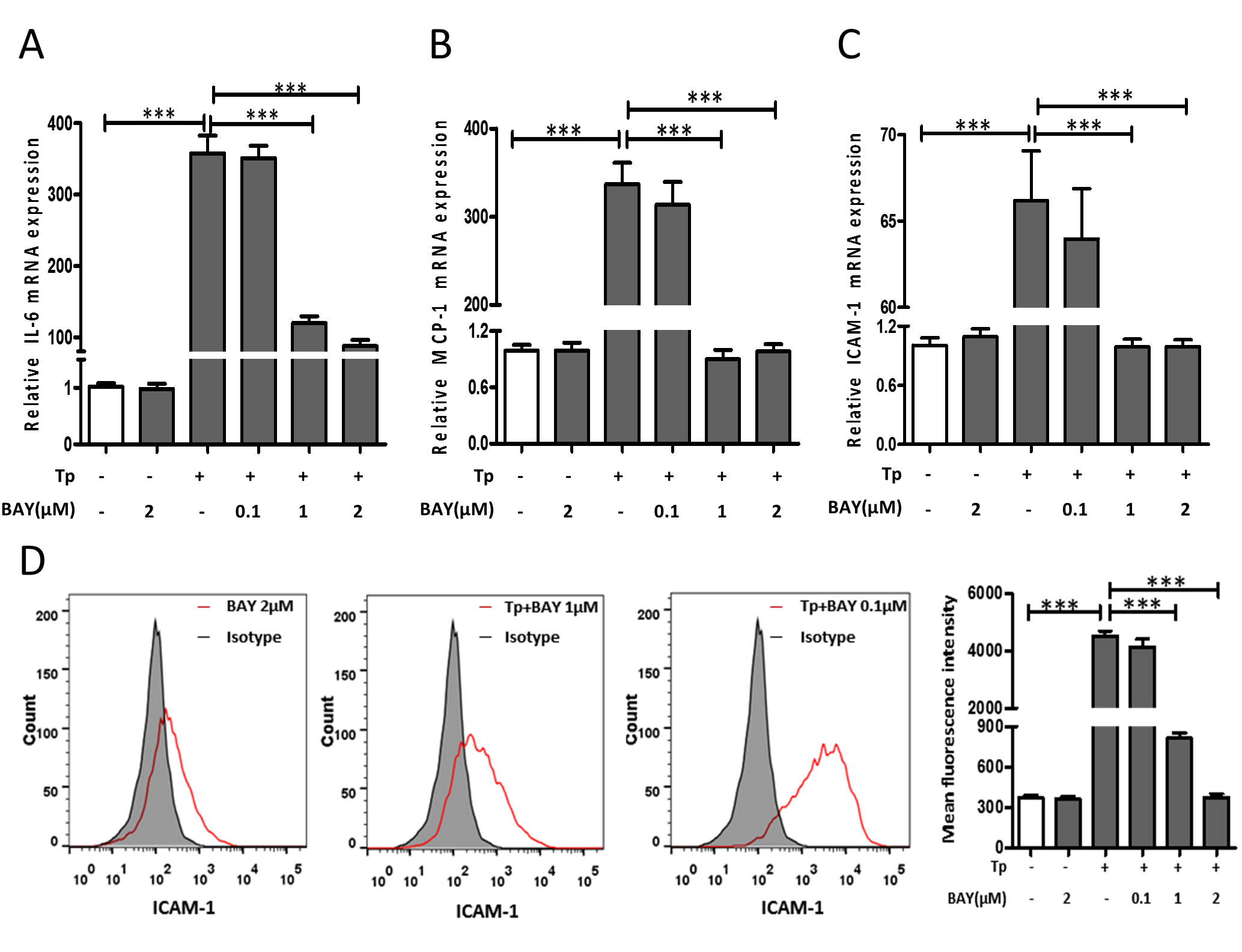

Supplement: Figure S3 — NF-κB signaling pathway components were essential for the induction of IL-6, MCP-1, and ICAM-1 expression by T. pallidum. HDVSMCs were pretreated with BAY11-7082 (2 μmol/L) for 1 h and then incubated with T. pallidum at an MOI of 100:1 for 24 h. The mRNA expression was evaluated by qRT-PCR. The MFI of ICAM was detected by flow cytometry. (A) The mRNA expression of IL-6. (B) The mRNA expression of MCP-1. (C) The mRNA expression of ICAM-1. (D) The MFI of ICAM-1. The values are the means ± SDs of experimental triplicates and are representative of the results of three independent experiments. Tp, T. pallidum; ICAM-1, intercellular cell adhesion molecule-1; BAY, BAY11-7082; μM, μmol/L; MFI, mean fluorescence intensity (***P < 0.001). [file Image_3.TIF]
